# Graphene oxide conjugated with polymers: a study of culture condition to determine whether a bacterial growth stimulant or an antimicrobial agent?

**DOI:** 10.1186/s12951-017-0328-8

**Published:** 2018-01-10

**Authors:** Ping-Ching Wu, Hua-Han Chen, Shih-Yao Chen, Wen-Lung Wang, Kun-Lin Yang, Chia-Hung Huang, Hui-Fang Kao, Jui-Cheng Chang, Chih-Li Lilian Hsu, Jiu-Yao Wang, Ting-Mao Chou, Wen-Shuo Kuo

**Affiliations:** 1 Department of Biomedical Engineering, National Cheng Kung University, Tainan 701, Taiwan, ROC; 2 Department of Food Science, National Penghu University of Science and Technology, Penghu 880, Taiwan, ROC; 3 Department of Internal Medicine, National Cheng Kung University Hospital, College of Medicine, National Cheng Kung University, Tainan 701, Taiwan, ROC; 4 Athena Institute of Holistic Wellness, Wuyishan, 354300 Fujian China; 5 Metal Industries Research & Development Centre, Kaohsiung 811, Taiwan, ROC; 6 Department of Materials Science Engineering, National Cheng Kung University, Tainan 701, Taiwan, ROC; 7 Department of Nursing, National Tainan Junior College of Nursing, Tainan 700, Taiwan, ROC; 8 Department of Chemical Engineering, National Cheng Kung University, Tainan 701, Taiwan, ROC; 9 Department of Microbiology & Immunology, National Cheng Kung University Hospital, College of Medicine, National Cheng Kung University, Tainan 701, Taiwan, ROC; 10 Department of Pediatrics, National Cheng Kung University Hospital, College of Medicine, National Cheng Kung University, Tainan 701, Taiwan, ROC; 11 Division of Plastic Surgery, Department of Surgery, E-Da Hospital, Kaohsiung 824, Taiwan, ROC; 12 Center for Micro/Nano Science and Technology, National Cheng Kung University, Tainan 701, Taiwan, ROC; 13 Advanced Optoelectronic Technology Center, National Cheng Kung University, Tainan 701, Taiwan, ROC

**Keywords:** Graphene oxide, Polyoxyalkyleneamine, Chitosan, Gram-negative bacteria, Gram-positive bacteria, Oxidative stress, Biofilm

## Abstract

**Background:**

The results showed that the deciding factor is the culture medium in which the bacteria and the graphene oxide (GO) are incubated at the initial manipulation step. These findings allow better use of GO and GO-based materials more and be able to clearly apply them in the field of biomedical nanotechnology.

**Results:**

To study the use of GO sheets applied in the field of biomedical nanotechnology, this study determines whether GO-based materials [GO, GO-polyoxyalkyleneamine (POAA), and GO-chitosan] stimulate or inhibit bacterial growth in detail. It is found that it depends on whether the bacteria and GO-based materials are incubated with a nutrient at the initial step. This is a critical factor for the fortune of bacteria. GO stimulates bacterial growth and microbial proliferation for Gram-negative and Gram-positive bacteria and might also provide augmented surface attachment for both types of bacteria. When an external barrier that is composed of GO-based materials forms around the surface of the bacteria, it suppresses nutrients that are essential to microbial growth and simultaneously produces oxidative stress, which causes bacteria to die, regardless of whether they have an outer-membrane-Gram-negative-bacteria or lack an outer-membrane-Gram-positive-bacteria, even for high concentrations of biocompatible GO-POAA. The results also show that these GO-based materials are capable of inducing reactive oxygen species (ROS)-dependent oxidative stress on bacteria. Besides, GO-based materials may act as a biofilm, so it is hypothesized that they suppress the toxicity of low-dose chitosan.

**Conclusion:**

Graphene oxide is not an antimicrobial material but it is a general growth enhancer that can act as a biofilm to enhance bacterial attachment and proliferation. However, GO-based materials are capable of inducing ROS-dependent oxidative stress on bacteria. The applications of GO-based materials can clearly be used in antimicrobial surface coatings, surface-attached stem cells for orthopedics, antifouling for biocides and microbial fuel cells and microbial electro-synthesis.

**Electronic supplementary material:**

The online version of this article (10.1186/s12951-017-0328-8) contains supplementary material, which is available to authorized users.

## Background

Because nanoscience produces new materials that are beneficial to human life, there is great interest in developing these materials for nanomedicine to eliminate malignant tumors and pathogenic microbes. Currently, many materials serve as antimicrobial agents: particles based on titanium oxide, silver, zinc oxide, magnesium oxide, cooper oxide, cadmium selenide/telluride, carbon nanotubes, and graphene-based materials [1–3]. Graphene is a two-dimensional monolayer of graphite with tightly bonded carbon atoms that are organized into a hexagonal lattice. Graphene has excellent conductivity, and good thermal, optical, and mechanical properties. Although graphene and graphene-based materials are being applied in almost every field, there is an unresolved debate [4–12] about their antimicrobial properties.

Bacteria are single-celled microbes and comprise a large range of prokaryotic microorganisms. Their cellular structure is simpler than that of other organisms because they have no nucleus or membrane-bound organelles. Bacteria are classified into several groups based on their diverse basic shapes (spheres, rods, and spirals) and sizes (ranging from 0.5 to 5 μm long). Some studies [5, 7, 11] report that graphene materials are antimicrobial, but others [8, 10, 12] report that they are not toxic to bacteria. To determine whether graphene-based materials exhibit antimicrobial effects, this study uses GO, GO-POAA, and GO-chitosan to determine their antimicrobial ability in this study. The results showed that the deciding factor is the culture medium in which the bacteria and the GO are incubated at the initial manipulation step, which is not discussed in previous studies [4–12]. When Gram-negative and Gram-positive bacteria are incubated in a nutrient medium with less than 50 μg mL^−1^ of GO, the bacteria proliferated so at this concentration, at least, GO-POAA is not a bactericide nor a growth inhibitor [8]. However, at concentrations of GO-POAA of more than 50 μg mL^−1^, the bacteria do not show the expected level of proliferation. In contrast, a concentration of 10 μg mL^−1^ GO-chitosan protects the bacteria from chitosan’s antibacterial properties. Most of the GO sheets and GO-based materials exhibit good antimicrobial activity only when they have an outer-membrane-Gram-negative-bacteria or when the outer-membrane-Gram-positive-bacteria are incubated with phosphate buffered saline (PBS) instead of some other nutrient.

## Results and discussion

### Characterization of GO sheets and GO-based materials

**Fig. 1:**
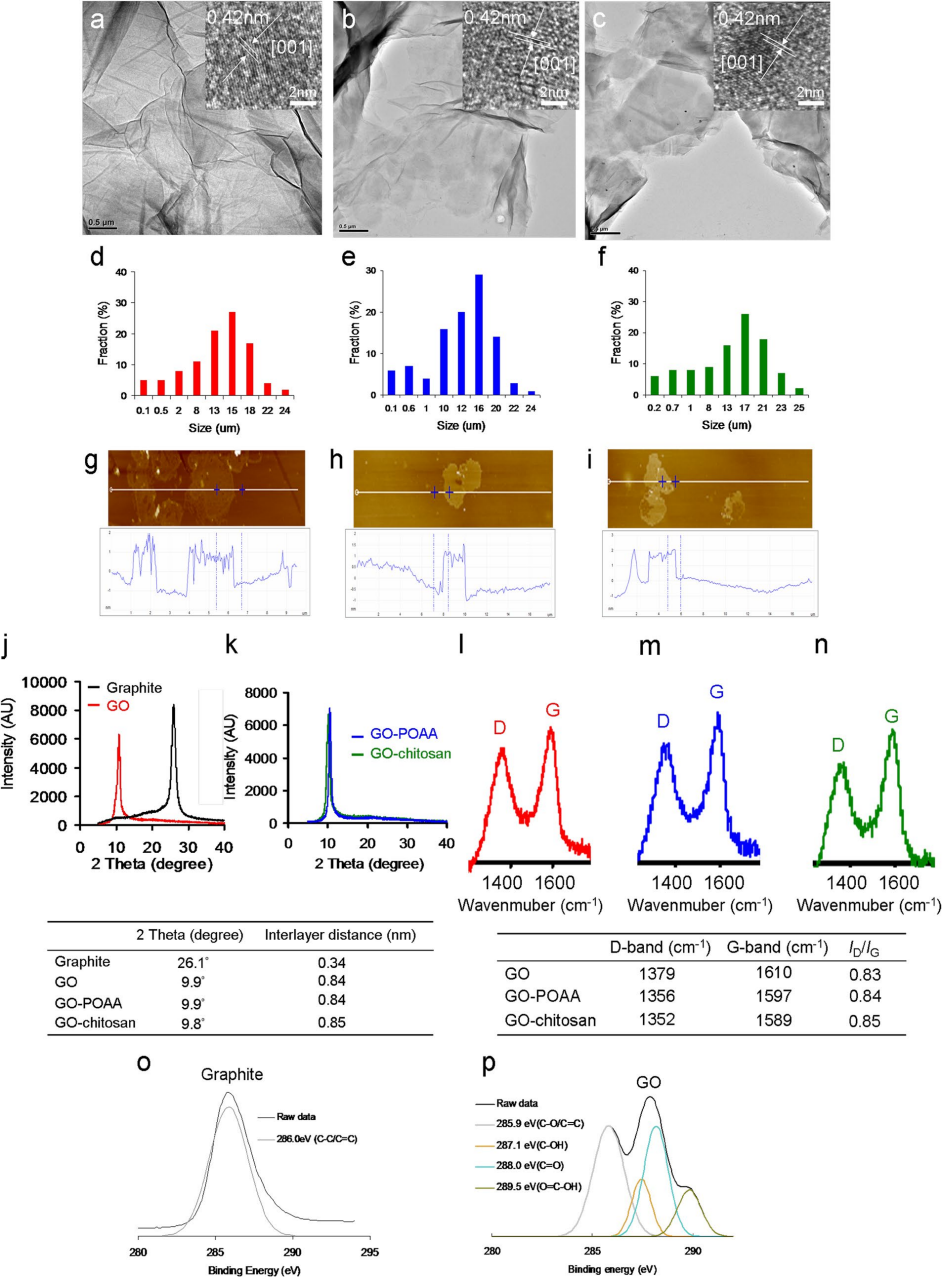
TEM images of the folded **a** GO, **b** GO-POAA and **c** GO-chitosan. The size is up to 20 μm wide (inset: interlayer distance of 0.34 nm from the image of high-resolution TEM), which corresponds to the size fractions of **d** GO, **e** GO-POAA and **f** GO-chitosan. The AFM image of **g** GO on mica, and the height difference between two arrows (the GO and mica) is 1.2 nm, consistent with the thickness of a single of layer GO sheet. The one-layer thickness of **h** GO-POAA and **i** GO-chitosan respectively increase to 1.5 and 1.7 nm. The XRD analysis to analyze the crystallinity. After the calculation, the respective interlayer distance for **j** pristine graphite, GO, **k** GO-POAA, and GO-chitosan are 0.34, 0.84, 0.84 and 0.85 nm. Raman spectroscopy is used to further determine the crystallinity of **l** GO, **m** GO-POAA and **n** GO-chitosan. The D and G bands are attributed to the local defects/disorders and the sp^2^ graphitized structure. The *I*_D_/*I*_G_ respective intensity ratios for GO, GO-POAA, and GO-chitosan are 0.83, 0.84 and 0.85. A larger ratio indicates more defects or disorders in a graphitized structure. XPS was used to examine the changes in the chemical states of **o** graphite and **p** GO. The deconvoluted C(1s) XPS spectra and fitted peaks: C–C/C=C for graphite, C–C/C=C, C–OH, C=O, and O–C–OH for GO. The C(1s) spectrum for graphite has no oxygen-contained functional groups, but that for GO exhibits these groups

A modified Hummers method was used to prepare GO from the same graphite sample [13]. transmission electron microscopy (TEM) images (Fig. 1a–c) show the mean lateral size of GO, GO-POAA, and GO-chitosan sheets to be up to 20 μm and the size fraction (Fig. 1d–f) measured using atomic force microscopy (AFM) (Fig. 1g–i), to be between 1 and 2 nm thick, which measurements are characteristic of a fully exfoliated GO sheet [14]. This was dried on a mica surface. The height profile diagram shows that the typical thickness of the observed single-layer GO sheet is around 1.1 ± 0.2 nm [14]. The typical thickness of GO is about a 0.4–0.7 nm increase in graphene thickness (~ 0.36 nm) because of the presence of epoxy, hydroxyl, and carboxyl groups on both sides of the oxide surface [14]. Therefore, a 1.2 nm in thickness corresponds to a one-layer GO sheet (Fig. 1g). In contrast, one-layer GO-POAA and GO-chitosan sheets are 1.5 and 1.7 nm thick, respectively, because the POAA and chitosan is successfully adsorbed on the surfaces of the GO sheets (Fig. 1h, i) [11, 15]. Because of the presence of exposed carboxylic acid after oxidation, the zeta potential shows the surface charge of the GO to be around − 30.9 mV. GO-POAA with negatively charged POAA adsorbed on the surface of the GO via electrostatic interaction has a surface charge of about 28.1 mV, and GO-chitosan, which uses the same linking strategy, has a surface charge of about 33.5 mV. X-ray diffraction (XRD), was used to analyze crystallinity. It shows a pristine graphite peaked (002) at about 26.3° (Fig. 1j), which gives an interlayer distance of 0.34 nm, and this disappears after oxidation. However, the GO material has a well-defined peak (001) at a diffraction angle near 2*θ* = 10.6°, which is indicative of good layer regularity with a repeating interlayer distance of 0.83 nm and confirms that the structure is well-ordered and lamellar. The increased basal spacing of GO is related to the accommodation of various oxygen species and water molecules and the changes in the carbon hexahedral grid plane [11, 16]. However, the diffraction angle is less than 10.6° when POAA is conjugated on the surface of GO (Fig. 1k). This increases the repeating interlayer distance. The phenomenon is also observed for GO-chitosan.

**Fig. 2:**
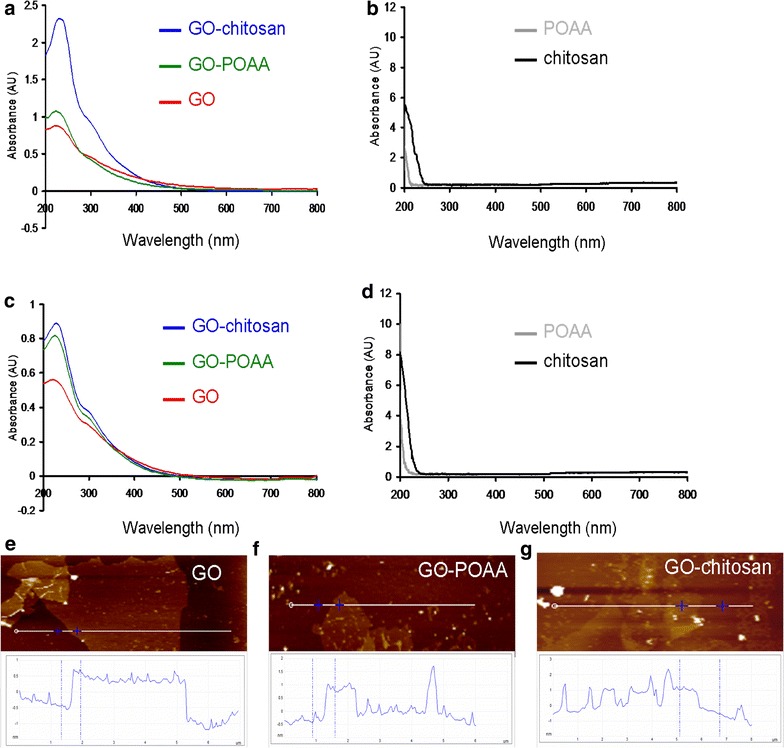
UV–vis spectra. For GO, GO-POAA, GO-chitosan, POAA and chitosan dissolved in **a**, **b** PBS and **c**, **d** nutrient medium. The AFM image of **e** GO on mica and the height difference between the two arrows (the GO and mica) is 1.16 nm, which is consistent with the thickness of a single layer GO (up to 20 μm wide). The one-layer thicknesses of **f** GO-POAA and **g** GO-chitosan respectively increased to 1.45 and 1.66 nm, respectively

Raman spectroscopy was also used to examine the crystallinity of the GO materials (Fig. 1l–n). Peaks occurred between 400 and 2000 cm^−1^. The major feature bands of GO are the so-called G band (~ 1610 cm^−1^), which comes from in-plane vibration of sp^2^ hybridized C–C bonds in a two dimensional hexagonal lattice and the D band, which corresponds to the defects, disorder, and sp^3^-hybridized carbon in graphene layers that break the translational symmetry of the lattice and occurs at about 1350 cm^−1^ (Fig. 1l). It is seen that the integrated intensity ratio of the D and G bands (*I*_D_/*I*_G_ ratio), which represents the degree of disorder, is 1.02, which indicates that graphite is successfully converted to GO. After the conjugation of POAA and chitosan, the position of the G band shifted from 1610 to 1587 cm^− 1^ (Fig. 1m, n), probably because the amino groups with the property of rich electron of POAA and chitosan are electron-donor molecules [17, 18], which cause high-frequency, tangential, vibrational modes in the carbon molecules in GO-based materials to shift to lower frequencies [19]. X-ray photoelectron spectroscopy (XPS) was also used to determine the surface chemistry of the graphite and GO materials (Fig. 1o, p and Table 1). Carbon atoms are the dominant element in these materials. The deconvoluted carbon spectra for GO are from a non-oxygenated ring (C–C/C=C, 285.9 eV), C–OH bonds (287.1 eV), carbonyl (C=O, 288.0 eV), and carboxylate (O=C–OH, 289.5 eV), respectively (Fig. 1p), but the C(1s) spectrum of graphite has no oxygen-contained functional groups (C–C/C=C, 286.0 eV), which confirms that GO is successfully oxidized from graphite (Fig. 1o). More convincing evidence comes from the ultraviolet–visible (UV–vis) spectra. The GO shows peaks at 225 and 298 nm in PBS (a water-based salt solution containing sodium phosphate, potassium chloride, and potassium phosphate; the osmotic concentration of PBS just matched is just sufficient) (Fig. 2a) and 225 and 297 nm in nutrient (Fig. 2c). The spectrum for GO-POAA shows red-shifts in the absorption peaks at around 226, 303 nm in PBS and at 227 and 303 nm in nutrient. The characteristic peak of GO-chitosan also exhibits a red-shift to 228 and 305 nm in PBS and 228 and 306 nm in nutrient because of the presence of POAA and chitosan on GO sheets. The results also show that there is no aggregation of these materials in PBS alone (Fig. 2a, b) or nutrient medium (Fig. 2c, d). More convincing evidence is provided by AFM. Samples for AFM that were respectively dissolved in PBS (Fig. 1g–i) and nutrient medium (Fig. 2e–g) were obtained by placing a drop of the sample on a mica, followed by evaporation of the solvent in a vacuum desiccator. The height profile diagram shows the thickness and size of GO sheets and GO-based materials were determined. Figure 2e shows it to be 1.16 nm in thick, which corresponds to one layer GO. However, the results show that the one-layer thickness of GO-POAA and GO-chitosan substantially increases to 1.45 and 1.66 nm (Fig. 2f, g), respectively, because of the presence of the POAA and the fact that chitosan is successfully adsorbed on the surfaces of the GO sheets. Fourier transform infrared (FTIR) spectroscopy was also used to analyze the exposed functional groups of GO and GO-based materials (Additional file 1: Figures S1, S2). These characterizations confirm that the GO is successfully synthesized and well-decorated with POAA and chitosan, respectively, and there is no aggregation of these materials in the presence of PBS and nutrient medium.

**Table 1:** The atomic ratio of O(1s)/C(1s) and carbon bonding composition determined by the XPS for GO

Atomic ratio O(1s)/C(1s)	Carbon bonding composition (%)			
	C–C/C=C	C–O	C=O	O–C=O
43.2%	58	8	25	11

### The colony forming unit (CFU) counting method and growth curve for bacteria for the antimicrobial test

**Fig. 3:**
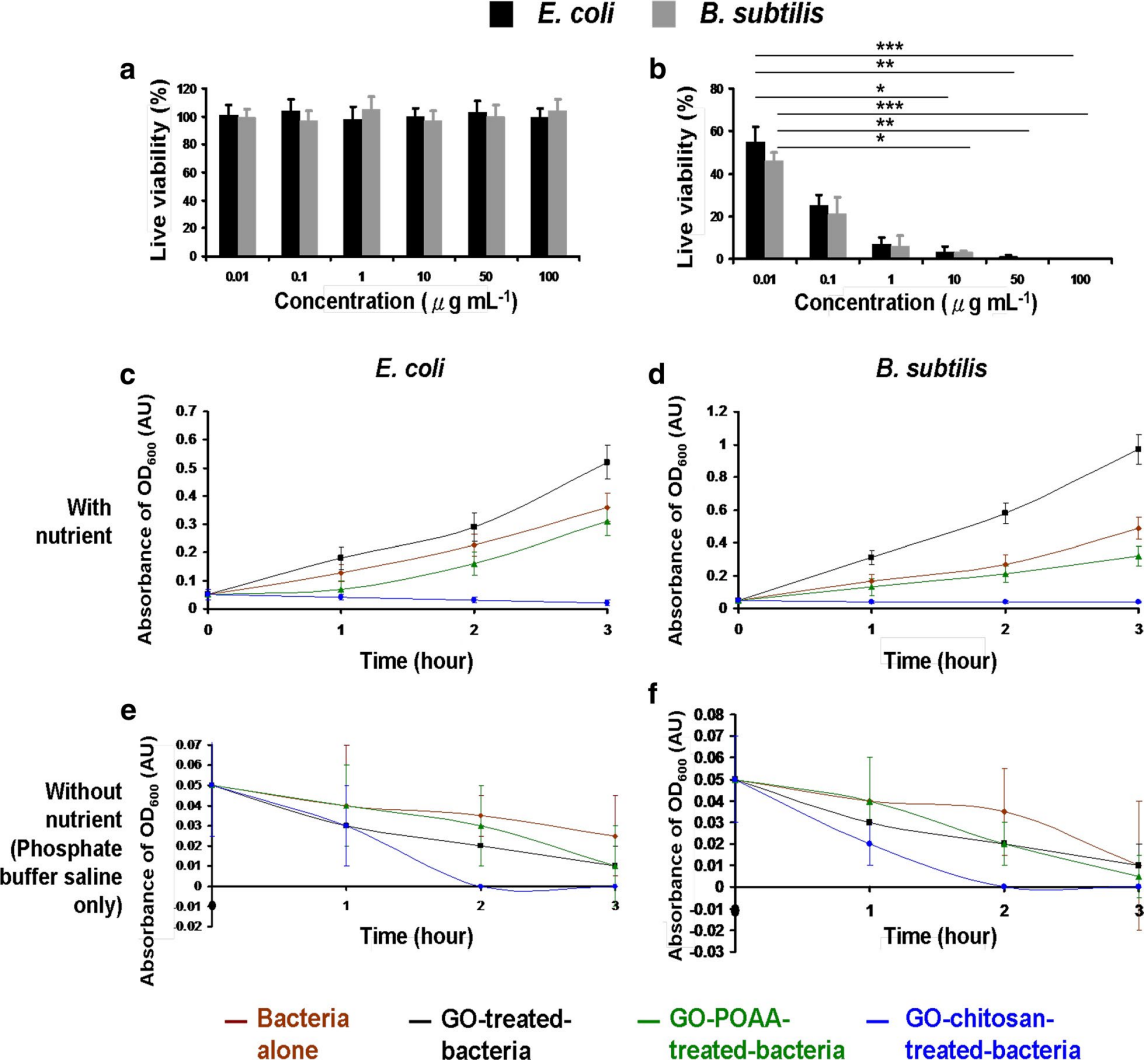
Bacterial viability. For **a**, **b** *E. coli* (OD_600_ ~ 0.05) and *B. subtilis* (OD_600_ ~ 0.05) that are treated with POAA and chitosan and incubated with **a** POAA and **b** chitosan to achieve the concentration of 0–100 μg mL^−1^ with nutrient medium for 3 h at 37 °C. The incubated bacteria were then diluted to a dilution factor of 10^−5^–10^−8^ and plated on the agar plates. The plates were incubated aerobically at 37 °C for 12–16 h. After incubation, the number of surviving bacteria was measured using by colony forming unit (CFU) counting assay was determined and switched the unit to percentage (%) which corresponds to the unit of CFU mL^−1^ (***p < 0.0001, **p < 0.001 and *p < 0.01 obtained by Student’s *t*-test), **c**–**f** Growth curves for bacteria. GO, GO-POAA, and GO-chitosan (GO materials, up to 20 μm wide, were delivered in a dose of 50 μg mL^−1^) were added to (c,e) *E. coli* (OD_600_ ~ 0.05), and **d**, **f** *B. subtilis* (OD_600_ ~ 0.05) and then incubated with either **c**, **d** nutrient or **e**, **f** PBS alone at 37 °C for 3 h. The absorbance at an optical density of 600 nm was recorded. Data are mean ± SD (n = 6)

The primary goal of this work is to determine whether GO and GO-based materials are antimicrobial to allow biomedical application of GO materials in the future. Consequently, Gram-negative *Escherichia coli* (*E. coli*), and Gram-positive *Bacillus subtilis* (*B. subtilis*) were the experimental templates. Broadly speaking, they differ in terms of cell-wall structure and because the former does not retain crystal violet dye but the latter does. To determine whether GO is antibacterial, POAA was conjugated and showed good biocompatibility (Fig. 3a) with GO as determined by the CFU counting method. This determines whether POAA reduces GO’s bactericidal capability [the number of surviving bacteria was determined and is expressed as a percentage (%) that corresponds to the unit of CFU mL^−1^]. In contrast, chitosan, which has a broad antimicrobial spectrum that includes both Gram-negative and Gram-positive bacteria (Fig. 3b) [20–22], was coated on the surface of GO to determine whether GO decreases chitosan’s bactericidal capability. It is hypothesized that chitosan is biocompatible with GO. In the antimicrobial test, the growth levels of the bacteria treated with GO-based materials were first monitored by measuring their absorbance at 600 nm. When they had been incubated for 3 h with a nutrient, the absorbance (at 600 nm) of *E. coli* and *B. subtilis*, which is initially 0.05 OD_600_, *E. coli* reaches 0.36 OD_600_ and that of *B. subtilis* reached 0.49 OD_600_ (Fig. 3c, d). Surprisingly, the OD_600_ peaked at 0.52 for *E. coli* and at 0.97 for *B. subtilis* when there is treatment of GO. However, the OD_600_ values for bacteria that are treated with GO-POAA peaked at 0.31 for *E. coli* and at 0.32 for *B. subtilis*. The OD_600_ values for bacteria that are treated with GO-chitosan decrease significantly to 0.02 for *E. coli* and to 0.04 for *B. subtilis*. As a result, when bacteria are treated with GO at 50 μg mL^−1^, they proliferated faster to a higher optical density than do cultures without GO, but their proliferation is inhibited when they are incubated with GO-POAA and GO-chitosan. In contrast, the OD_600_ values for GO-, GO-POAA- and GO-chitosan-treated-*E. coli* were respectively decrease to 0.01, 0.01, and 0 when incubated with PBS alone, and the OD_600_ values for *B. subtilis* are 0.01, 0.005, and 0 OD_600_, respectively (Fig. 3e, f). The results indicated that GO does not inhibit bacterial proliferation, but promotes it, and allows bacteria to proliferate even faster than if they are cultured with only a nutrient. GO-based materials appear to inhibit bacterial proliferation because they have a lower optical density. In contrast, the two experimental bacteria are always inhibited when they are treated with PBS and any type of GO sheets or GO-based materials.

### Characterization using with TEM and a further antimicrobial test

**Fig. 4:**
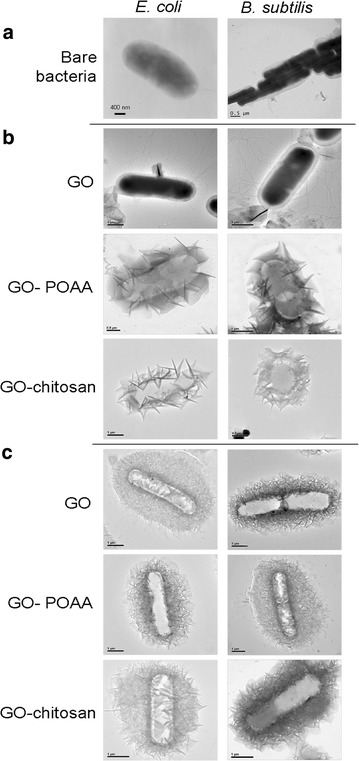
TEM images. Bacteria with or without GO, GO-POAA, and GO-chitosan are characterized by TEM: **a** bare *E. coli* and *B. subtilis* without any treatment. The formation of an external barrier of GO sheets and GO-based materials on the surfaces of these two bacteria after 30 min of incubation with **b** either nutrient **c** or PBS, respectively. (GO materials, up to 20 μm wide, were delivered in a dose of 50 μg mL^−1^)

**Fig. 5:**
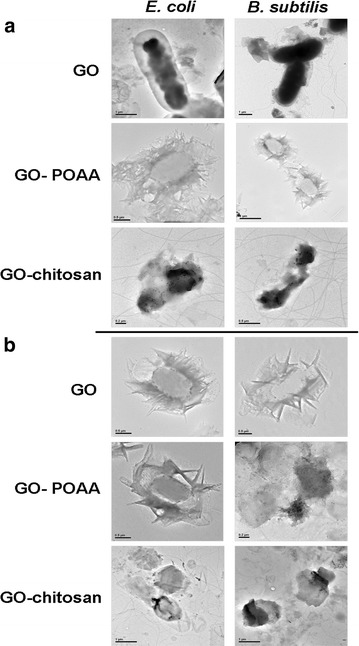
TEM images. Bacteria with or without GO, GO-POAA, and GO-chitosan are characterized by TEM: the formation of an external barrier of GO sheets and GO-based materials on the surfaces of these two bacteria after 3 h of incubation with **a** either nutrient **b** or PBS, respectively. (GO materials, up to 20 μm wide, were delivered in a dose of 50 μg mL^−1^)

**Fig. 6:**
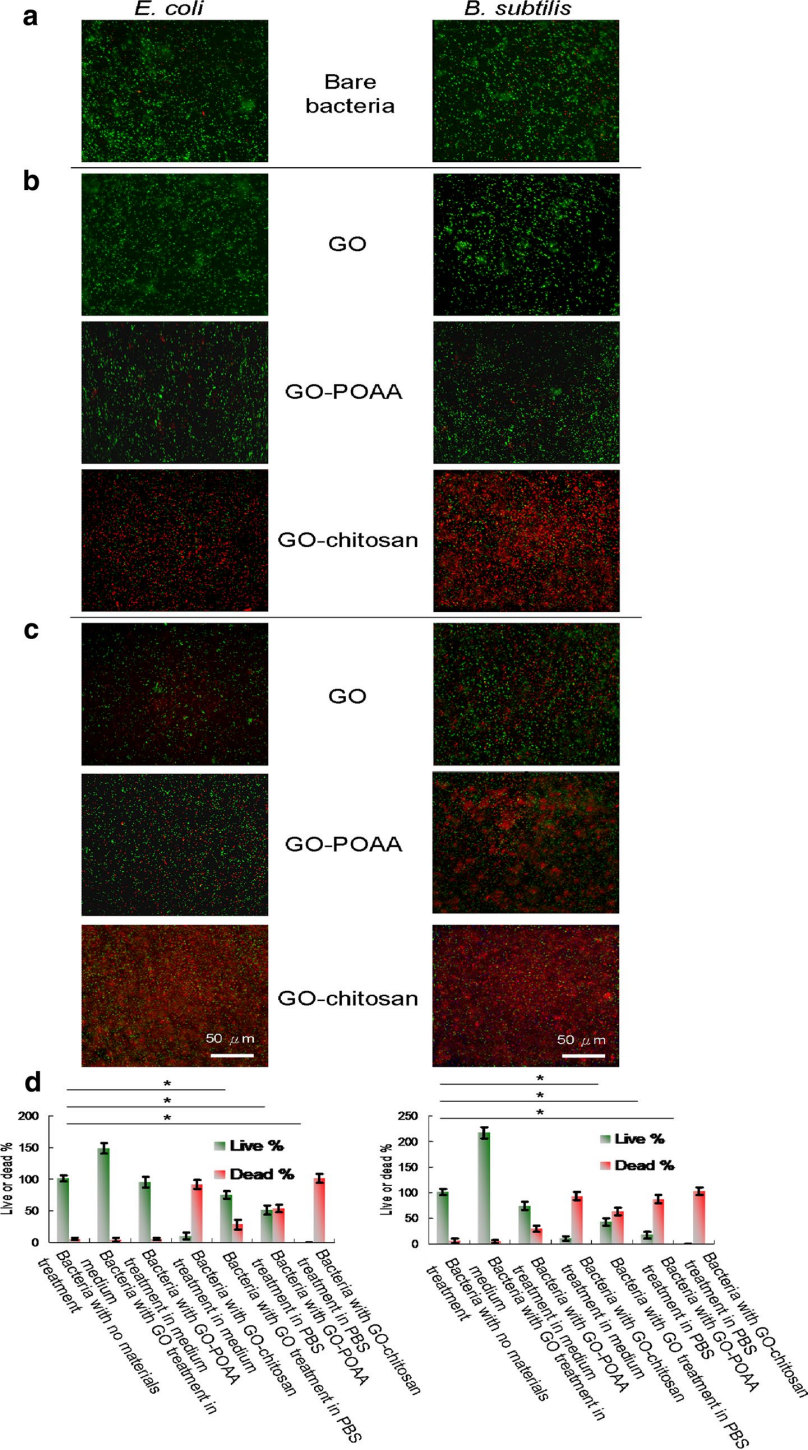
Bacterial viability shown by fluorescence and quantification. Images and viabilities of **a** bare *E. coli* and *B. subtilis* after treatment with GO, GO-POAA, and GO-chitosan and 3 h of incubation with **b** either nutrient **c** or PBS. The bacteria were stained using a LIVE/DEAD kit to obtain the fluorescent images, and **d** quantified for viability was estimated (GO materials, up to 20 μm wide, were delivered in a dose of 50 μg mL^−1^). Negative control (0 μg mL^−1^): bare bacteria without any treatment. For the live % for both bacteria treated with PBS, *p* = 0.832 and *p* = 0.419 are the group of bacteria with no materials treatment and bacteria with GO treatment in PBS; *p* = 0.325 and *p* = 0.097 are the group of bacteria with no materials treatment and bacteria with GO-POAA treatment in PBS; *p* < 0.001 and *p* < 0.001 are the group of bacteria with no materials treatment and bacteria with GO-chitosan treatment in PBS. Data are mean ± SD (n = 6). *p value obtained by Student’s *t*-test

Furthermore, TEM was used to check the images of GO-treated bacteria and GO-based material-treated bacteria. Incubating negatively charged *E. coli* (OD_600_ ~ 0.05) and *B. subtilis* (OD_600_ ~ 0.05) respectively (Fig. 4a) to with negatively charged GO sheets and positively charged GO-based materials (GO materials were delivered in a dose of 50 μg mL^−1^) in a nutrient medium at 37 °C for 30 min results in only a few and small GO sheets being adsorbed on both bacterial surfaces, but GO-POAA and GO-chitosan are present in greater amounts on the surfaces because of the electrostatic interaction (Fig. 4b). Large amounts of GO sheets and GO-based materials are densely formed and surrounded on the bacterial surface when nutrient medium is replaced with PBS and incubated for 30 min at 37 °C (Fig. 4c). Bacteria must filter external ions and assimilate nutrition via the cell wall to maintain and develop their physiological functions, so bacteria that are incubated with PBS are starved of nutrition, and GO and GO-based materials are absorbed and formed in the external barrier on the bacterial surface [23]. TEM imaging clearly shows whether any material interacts with the bacteria [14–16]. The TEM images of the bacteria after 3 h of incubation also showed no apparent increase in the number of attached GO sheets on bacteria that is incubated in a nutrient medium, which indicates normal live bacterial morphology (Fig. 5a). The GO-POAA-treated *E. coli* shows no exceptional morphology, but the GO-POAA-treated *B. subtilis* changes shape slightly, so GO-POAA begins to exhibit a bacteriostatic or bactericidal ability when incubated with a nutrient. Bacteria that is treated with GO-chitosan also shows GO-chitosan with severe damage. However, all of the GO-treated and GO-based-materials that are used to treat bacteria with PBS alone (Fig. 5b) developed a more abnormal morphology than those that are treated with a nutrient medium. In summary, Gram-positive and Gram-negative bacteria that are treated with GO, but not with GO-based materials, proliferated. Figures 4, 5 also show that when an external barrier is formed, bacteria are unable to function normally and they begin to die. The viability of bacteria is shown by fluorescence and quantification (Fig. 6) [14]. The bare bacteria of *E. coli* and *B subtilis* exhibited almost no damage, as shown by the predominance of green fluorescence, which indicates live bacteria (Fig. 6a). However, the number of dead bacteria is significant for treatment with GO materials, as indicated by the predominance of red fluorescence (Fig. 6b, c). The results of further antimicrobial test that are shown in Fig. 6b, c, quantify the bacterial viability. After treatment, both GO-treated-bacteria are at least 1.5 times more viable than bacteria alone for the test at 50 μg mL^−1^ with a nutrient (Fig. 6d). However, the viability of both bacteria treated with GO-POAA decreases to at least 75%. The viability decreases to less than 10% when the GO-chitosan is treated. In contrast, treatment with GO, GO-POAA and GO-chitosan gives significantly less bacterial proliferation and viability than treatment with only PBS.

**Fig. 7:**
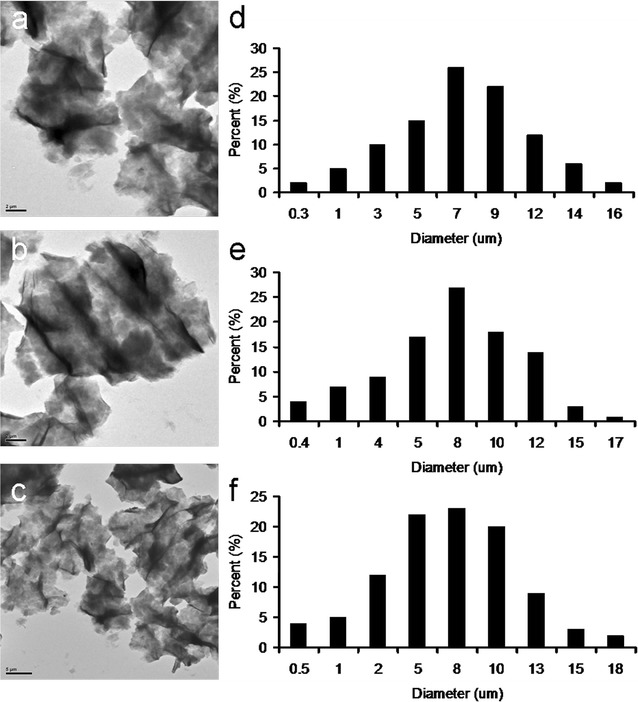
TEM images. The folded **a** GO, **b** GO-POAA and **c** GO-chitosan which correspond to the size fractions of **d** GO, **e** GO-POAA and **f** GO-chitosan. The mean lateral size is up to 10 μm

The qualitative CFU counting method, which shows the effect of GO sheets and GO-based materials on the proliferation of microorganisms, was also used to determine the proliferation of bacteria that are treated with GO-based materials [14, 16]. Bacterial viability was determined after treating *E. coli* and *B. subtilis* with a range of GO and GO-based materials (10–300 μg mL^−1^). The number of surviving bacteria was determined and is expressed as a percentage (%) that corresponds to the unit of CFU mL^−1^ (Tables 2, 3). For the tests at 10 and 50 μg mL^−1^ with a nutrient, both GO-treated-bacteria have at least 1.5 times the viability of bacteria alone. For 50 μg mL^−1^ test, the survival rate was determined using a LIVE/DEAD kit [14] (Fig. 6d), which gives the same results as determining by the CFU counting method. Even at 200 μg mL^−1^, the biocompatibility of GO is still good, but it begins to decrease when the dose reached 300 μg mL^−1^. These results showed that GO is a general growth enhancer that can act as a scaffold for bacterial attachment and proliferation, not a bactericidal or bacteriostatic material. However, the viability of both bacteria that are treated with GO-POAA is at least 1.3 times that for bacteria that are only cultured with a nutrient at 10 μg mL^−1^. The viability decreases to 0 when the GO concentration is increased. The electrostatic interaction between the negatively charged GO and positively charged POAA causes the formation of an external barrier that suppresses nutrients that are essential to microbial growth and promotes changes in the properties of membrane wall permeability. This change in turn promotes internal osmotic imbalances and inhibits the growth of microorganisms [23, 24]. This is shown in Figs. 4b and 5a. However, the proliferation of both bacteria is inhibited when they are incubated with GO-chitosan, but GO, at a dose of 10 μg mL^−1^, reduces chitosan’s bactericidal capability. In contrast, treatment with GO and with GO-POAA significantly reduces bacterial proliferation and viability over treatment with only PBS. The viability of bacteria that are treated with GO is size-dependent [5–12]. TEM images (Fig. 7a–c) show smaller GO, GO-POAA, and GO-chitosan sheets (the mean lateral size is less than 10 μm wide, Fig. 7d–f), and there is a similar trend for the viability of smaller GO-based-treated-*E. coli* and -*B. subtilis* as that shown in Tables 2, 3: they were not susceptible to the size of GO sheets or GO-based materials (Tables 4, 5). In summary, GO and GO-based materials’ bactericidal capability results from the formation of an external barrier that prevents bacteria from assimilating nutrition and from undertaking migration that leads to death and from the ionic surface interaction that causes cell wall leakage in PBS [23, 24]. In other words, the deciding factor for determining the antimicrobial ability of GO sheets is the culture medium in which the bacteria and the GO are incubated at the initial manipulation step. Materials would form an external barrier around the bacterial surface that promotes the microbial growth when only PBS is used, but no obviously external barrier is formed when they are incubated with a nutrient. Using GO-based materials suggested promotes changes in the properties of the permeability of the membrane wall, which causes internal osmotic imbalances and inhibits the growth of microorganism in both culture media.

**Table 2:** Bacterial viability

Incubate with MB medium				Incubate with PBS buffer			
Concentration (μg/mL)	Viability (%) with treatment of GO	Viability (%) with treatment of GO-POAA	Viability (%) with treatment of GO-chitosan	Concentration (μg/mL)	Viability (%) with treatment of GO	Viability (%) with treatment of GO-POAA	Viability (%) with treatment of GO-chitosan
10	214 ± 10	132 ± 13	36 ± 11	10	104 ± 7	98 ± 12	18 ± 10
50	152 ± 13	96 ± 12	8 ± 6	50	73 ± 10	53 ± 9	0
100	104 ± 6	30 ± 8	0	100	11 ± 8	5 ± 2	0
200	95 ± 12	0	0	200	0	0	0
300	71 ± 9	0	0	300	0	0	0

Measured by treating GO and GO-based materials (up to 20 μm wide) with *E. coli*. 10–300 μg mL^−1^ of materials were treated with *E. coli*. After 3 h incubation with nutrient medium or PBS, respectively, the CFU counting method was used. The number of surviving bacteria was determined and is expressed as a percentage (%) that corresponds to the unit of CFU mL^−1^. Data are mean ± SD (n = 6)

**Table 3:** Bacterial viability

Incubate with MB medium				Incubate with PBS buffer			
Concentration (μg/mL)	Viability (%) with treatment of GO	Viability (%) with treatment of GO-POAA	Viability (%) with treatment of GO-chitosan	Concentration (μg/mL)	Viability (%) with treatment of GO	Viability (%) with treatment of GO-POAA	Viability (%) with treatment of GO-chitosan
10	278 ± 14	189 ± 9	26 ± 12	10	112 ± 15	68 ± 12	13 ± 5
50	223 ± 15	72 ± 13	7 ± 3	50	40 ± 7	19 ± 4	0
100	96 ± 12	18 ± 7	0	100	3 ± 1	2 ± 1	0
200	87 ± 10	6 ± 2	0	200	0	0	0
300	45 ± 8	0	0	300	0	0	0

Measured by treating GO sheets and GO-based materials (up to 20 μm wide) with *B. subtilis*. 10–300 μg mL^−1^ of materials were treated with *B. subtilis*. After 3 h incubation with nutrient medium or PBS, respectively, the CFU counting method was used. The number of surviving bacteria was determined and is expressed as a percentage (%) that corresponds to the unit of CFU mL^−1^. Data are mean ± SD (n = 6)

**Table 4:** Bacterial viability

Incubate with nutrient medium				Incubate with PBS buffer			
Concentration (μg/mL)	Viability (%) with treatment of GO	Viability (%) with treatment of GO-POAA	Viability (%) with treatment of GO-chitosan	Concentration (μg/mL)	Viability (%) with treatment of GO	Viability (%) with treatment of GO-POAA	Viability (%) with treatment of GO-chitosan
10	208 ± 13	137 ± 11	43 ± 9	10	110 ± 13	93 ± 10	10 ± 5
50	161 ± 12	95 ± 10	13 ± 4	50	68 ± 8	38 ± 6	0
100	107 ± 12	22 ± 7	0	100	19 ± 7	12 ± 6	0
200	85 ± 8	6 ± 3	0	200	0	4 ± 3	0
300	66 ± 7	3 ± 1	0	300	0	0	0

Measured by treating smaller GO sheets and GO-based materials (up to 10 μm wide) with *E. coli*. 10–300 μg mL^−1^ of materials were treated with *E. coli*. After 3 h incubation with nutrient medium or PBS, respectively, the CFU counting method was used. The number of surviving bacteria was determined and is expressed as a percentage (%) that corresponds to the unit of CFU mL^−1^. Data are mean ± SD (n = 6)

**Table 5:** Bacterial viability

Incubate with nutrient medium				Incubate with PBS buffer			
Concentration (μg/mL)	Viability (%) with treatment of GO	Viability (%) with treatment of GO-POAA	Viability (%) with treatment of GO-chitosan	Concentration (μg/mL)	Viability (%) with treatment of GO	Viability (%) with treatment of GO-POAA	Viability (%) with treatment of GO-chitosan
10	255 ± 13	158 ± 7	18 ± 7	10	111 ± 9	81 ± 10	6 ± 2
50	194 ± 11	65 ± 9	3 ± 1	50	43 ± 13	26 ± 8	0
100	95 ± 9	10 ± 6	0	100	4 ± 1	3 ± 2	0
200	93 ± 13	0	0	200	0	0	0
300	53 ± 10	0	0	300	0	0	0

Measured by treating smaller GO sheets and GO-based materials (up to 10 μm wide) with *B. subtilis*. 10–300 μg mL^−1^ of materials were treated with *B. subtilis*. After 3 h incubation with nutrient medium or PBS, respectively, the CFU counting method was used. The number of surviving bacteria was determined and is expressed as a percentage (%) that corresponds to the unit of CFU mL^−1^. Data are mean ± SD (n = 6)

### Assay of oxidative stress

**Fig. 8:**
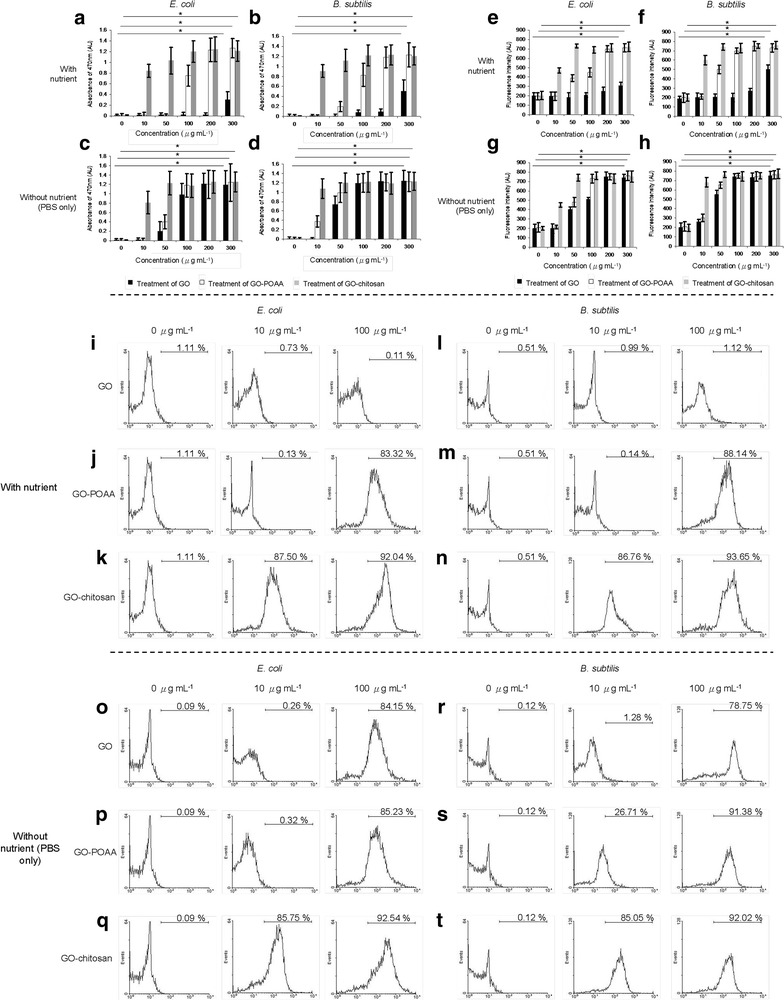
The ROS assays. After **a**, **c** *E. coli* and **b**, **d** *B. subtilis* were treated with GO, GO-POAA, and GO-chitosan (up to 20 μm wide) in either **a**, **b** nutrient or **c**, **d** PBS alone at 37 °C for 3 h, superoxide radical anion (O_2_^**·**−^) were generated. XTT was used to monitor the generated superoxide radical anion and the absorbance at 470 nm was recorded. Singlet Oxygen Sensor Green Reagent was used to directly detect singlet oxygen. Singlet oxygen (^1^O_2_) measurements were conducted by monitoring similarly treated **e**, **g**—*E. coli* and **f**, **h**—*B. subtilis* in either **e**, **f** nutrient or **g**, **h** PBS alone with the same treatment. For *E. coli*, **a** *p* = 0.266, *p* < 0.001 and *p* < 0.001, and **c** *p* < 0.001, *p* < 0.001 and *p* < 0.001 are for treatment with GO, GO-POAA and GO-chitosan, respectively; for *B. subtilis*, **b** *p* = 0.179, *p* < 0.001 and *p* < 0.001, and **d** *p* < 0.001, *p* < 0.001 and *p* < 0.001 are for treatment of GO, GO-POAA and GO-chitosan, respectively; for *E. coli*, **e** *p* = 0.845, *p* = 0.337 and *p* = 0.341, and **g** *p* = 0.297, *p* = 0.281 and *p* = 0.266 are for treatment of GO, GO-POAA and GO-chitosan, respectively; for *B. subtilis*, **f** *p* = 0.415, *p* = 0.360 and *p* = 0.329, and **h** *p* = 0.305, *p* = 0.311 and *p* = 0.282 are for treatment of GO, GO-POAA and GO-chitosan, respectively. Data are mean ± SD (n = 6). *p value obtained by Student’s *t*-test. CM-H_2_DCFDA was used to detect the generated hydroxyl peroxide (H_2_O_2_) via flow cytometry. Measurements were conducted for similarly treated (**i**–**k**, **o**–**q**—*E. coli* and **l**–**n**, **r**–**t**—*B. subtilis* in either **i**–**n** nutrient or **o**–**t** PBS with the same treatment. The higher the percentage (histogram moves to the right), the greater is the oxidative stress induced. Negative control (0 μg mL^−1^): bacteria alone without any treatment

The cytotoxicity of multi-walled carbon nanotubes and single-walled carbon nanotubes operates using several mechanisms, such as physical damage that causes a rupture and oxidative stress [25–27]. To determine whether the oxidative stress that is generated by the ROS is involved in the antimicrobial activity of GO sheets and GO-based materials, ROS experiments were undertaken. Superoxide radical anion (O_2_^**·**−^), singlet oxygen (^1^O_2_), and hydrogen peroxide (H_2_O_2_) are three types of ROS that induce DNA damage, the oxidation of fatty acid amino acid, and enzyme inactivation, all of which lead to cell injury. Firstly, the intensity of superoxide radical anion and singlet oxygen were measured by monitoring the absorbance of 2, 3-bis (2-methoxy-4-nitro-5-sulfophenyl)-2H-tetrazolium-5-carboxanilide (XTT) [7, 28] at 470 nm and the fluorescence intensity from a Singlet Oxygen Sensor Green Reagent [16] after the bacteria were incubated with GO and GO-based materials in a range of dosages (10–300 μg mL^−1^). When GO-treated-bacteria are incubated with both culture media for 3 h, they do not induce apparent oxidative stress at low concentrations. When the GO concentration is higher, the more of these two ROS species (O_2_^**·**−^ and ^1^O_2_) are generated (Fig. 8a–h, respectively). GO and GO-based materials induced dose-dependent H_2_O_2_ [29] production (Fig. 8i–t). GO-POAA- and GO-chitosan-treated-bacteria are also more cytotoxic than did GO-treated bacteria. Glutathione (γ-l-glutamyl-l-cysteinyl-glycine, GSH), which is a thiol-tripeptide, can prevent damages to cellular components due to oxidation tress. The thiol group from GSH is oxidized to a disulfide bond, which converts GSH into glutathione disulfide [6, 15, 30]. In vitro GSH oxidation was also used to examine the generated O_2_^**·**−^ anions that are generated from GO- and GO-based materials (10–100 μg mL^−1^) for bacteria that are treated with both culture media for 3 h of incubation (Additional file 1: Figure S3) [6] and the oxidation is summarized in Tables 6, 7. The oxidation of GSH shows that these GO- and GO-based materials are capable of inducing O_2_^**·**−^ anion-dependent oxidative stress in bacteria. To prevent the possibility of O_2_^**·**−^ and ^1^O_2_ production if bacteria generated ROS after 30 min or 3 h of incubation, which could compromise the experiment, the following ROS experiments with bacteria alone were also conducted (Additional file 1: Figure S4). Expectedly, after conducting the experiments, non-ROS was generated on bacteria in the condition of medium or PBS for 30 min, as well as in medium for 3 h; the generated ROS slightly increased under the treatment of PBS for 3 h. In summary, the GO sheets and GO-based materials begin to show bacteriostatic or bactericidal ability when an external barrier forms around the bacterial surface. After the bacteria develop oxidative stress, they were treated with GO-based materials, which may have been responsible for the death of the bacteria.

**Table 6:** ROS assay

Incubate with MB medium				Incubate with PBS buffer			
Concentration (μg/mL)	Loss of GSH (%) of GO	Loss of GSH (%) of GO-POAA	Loss of GSH (%) of GO-chitosan	Concentration (μg/mL)	Loss of GSH (%) of GO	Loss of GSH (%) of GO-POAA	Loss of GSH (%) of GO-chitosan
0	0 ± 2.2	0 ± 1.5	0 ± 3.1	0	0 ± 3.3	0 ± 1.8	0 ± 2.9
10	0 ± 5.1	0 ± 4.3	59.4 ± 6.2	10	0 ± 1.4	4.8 ± 2.7	80.1 ± 6.2
100	0 ± 3.2	67.3 ± 3.9	99.5 ± 2.4	100	86.5 ± 4.8	93.4 ± 5.3	99.9 ± 3.4

In vitro GSH oxidation is used to determine the oxidation stress that is mediated by the GO- and GO-based materials (up to 20 μm wide) that are used to treat *E. coli*. Data are mean ± SD (n = 6)

**Table 7:** ROS assay

Incubate with MB medium				Incubate with PBS buffer			
Concentration (μg/mL)	Loss of GSH (%) of GO	Loss of GSH (%) of GO-POAA	Loss of GSH (%) of GO-chitosan	Concentration (μg/mL)	Loss of GSH (%) of GO	Loss of GSH (%) of GO-POAA	Loss of GSH (%) of GO-chitosan
0	0 ± 3.7	0 ± 2.0	0 ± 1.5	0	0 ± 1.2	0 ± 2.6	0 ± 3.3
10	0 ± 2.6	0 ± 3.9	78.6 ± 1.8	10	0 ± 2.6	30.6 ± 3.5	85.8 ± 2.9
100	5.5 ± 1.3	79.1 ± 5.2	98.9 ± 1.2	100	95.7 ± 3.9	99.2 ± 2.8	99.8 ± 1.1

In vitro GSH oxidation is used to determine the oxidation stress that is mediated by the GO- and GO-based materials (up to 20 μm wide) that are used to treat *B. subtilis*. Data are mean ± SD (n = 6)

## Conclusions

The debate concerning the antimicrobial activity of graphene-based materials is lengthy. This study determines the decisive point at which a the bactericidal or bacteriostatic ability is generated from GO sheets and GO-based materials. It is also determined whether the GO-treated-bacteria exhibit these characteristics for both Gram-positive and Gram-negative bacteria that are incubated in the environment of a nutrient. The results show that GO is not an antimicrobial material but also is a general growth enhancer that can act as a biofilm that allows bacterial attachment and proliferation. Other GO-based materials provide further evidence for the antimicrobial activity because they conjugate with biocompatible or antibacterial molecules. The conjugation of the positively charged POAA and chitosan with the negatively charged GO via electrostatic interaction, promotes changes in membrane wall permeability, which results in internal osmotic imbalances and inhibits the growth of microorganism. An external barrier also promotes suppression of essential nutrients that are essential to microbial growth and simultaneously produces oxidative stress. Although this results in the death of bacteria, GO-POAA still stimulates the increased growth of bacteria at low concentration of GO-based materials and GO-chitosan suppresses the toxicity of low-dose chitosan. The results also show that these GO- and GO-based materials can induce ROS-dependent oxidative stress in bacteria. These results allow better use of GO and GO-based materials in the field of biomedical nanotechnology, such as the design of graphene-based antimicrobial surface coatings, in facilitating surface-attached stem cells for orthopedics, applying in antifouling techniques for biocides and in microbial fuel cells and microbial electrosynthesis [31–34].

## Methods

### Materials

Graphite powder was purchased from Bay carbon, SP-1, USA. POAA was purchased from HUNTSMAN (USA). Chitosan, KBr, NaCl, KCl, Na_2_HPO_4_·2H_2_O, KH_2_PO_4_, phosphotungstate solution, catalase (from Bovine Liver), ethanol and XTT were purchased from Sigma-Aldrich Co. (USA) and Fluka (USA). NaNO_3_, microbiology powder and microbiology agar were purchased from Merck (Germany). H_2_SO_4_ was purchased from Wako (Japan). KMnO_4_ was purchased from J. T. Baker (USA). H_2_O_2_ was purchased from Shimakyu (Japan). Singlet Oxygen Sensor Green Reagent and 5-(and 6-)chloromethyl-2′,7′-dichlorodihydrofluorescein diacetate (CM-H_2_DCFDA) were purchased from Invitrogen (USA). All chemicals and reagents were of analytical grade.

### GO preparation

Graphene oxide was prepared from a natural graphite powder using a modified Hummers’ method [13]. The graphite powder (0.417 mol) and NaNO_3_ (0.0294 mol) were introduced to concentrated H_2_SO_4_ (18 M) in and ice-bath. KMnO_4_ (0.095 mol) was added gradually with stirring, so that the temperature of the mixture was kept below 20 °C. The mixture was then stirred at 35 °C for 4 h. De-ionized water was then slowly added to the mixture, followed by stirring the mixture at 98 °C for 15 min. The suspension was further diluted to 700 mL and stirred for 30 min. The reaction was terminated by adding H_2_O_2_ (35 wt%) with stirring at room temperature. The product was washed several times with de-ionized water were conducted and the GO specimen was obtained by drying the precipitate of the final slurry at 40 °C for 24 h.

### Characterization

Droplets of the bacteria were stained using 1% aqueous phosphotungstate solution for negative staining and the bacteria were allowed to dry on grids that were coated with Formvar. The bacteria alone and bacterial materials were then subject to TEM (JEOL 1400, at 80 kV; JEOL 2100, at 200 kV; and JEOL 3010, at 300 kV, Japan) observation. The droplet was then evaporated in a vacuum desiccator. Samples for AFM (multimode 8, Bruker, Germany) that were dissolved in PBS (Fig. 1g–i) and nutrient medium (Fig. 2e–g) were obtained by placing a drop of the sample on a mica, followed by evaporation of the solvent in a vacuum desiccator. The height profile diagram, thickness and size of GO sheets and GO-based materials were determined. The crystalline structures of graphene and GO were identified using XRD (Bruker AXS Gmbh, Germany/D2 Phaser) with Cuκα radiation (λ = 1.54060 Å) at 40 kv and 40 mA. The FTIR spectra for the GO sheets and GO-based materials were collected using a spectrometer (PerkinElmer RX1, USA) and the UV–vis absorption spectra were recorded using another spectrometer (U-4100, Hitachi, Japan). The data for zeta potential was measured using a spectrometer (Manern Nano-ZS90, UK). The crystallinity of GO was determined by exposure to a 532 nm laser via Raman spectroscopy (DXR, Thermo Scientific, USA). The surface chemistry of the graphite and GO-based materials were determined using by XPS (PHI 5000, VersaProbe). The function of each instrument is summarized in Additional file 1: Table S1.

### Synthesis and characterization of GO-based materials

To conjugate POAA and chitosan, positively charged POAA (100 μg mL^−1^) and chitosan (100 μg mL^−1^) were coated onto the surface of as-prepared GO sheets (50 μg mL^−1^) that had a negative charge of about − 30.9 mV due to electrostatic interaction. Consequentially, the GO-POAA that had a surface charge of about 28.1 mV and GO-chitosan that had a surface charge of about 33.5 mV were prepared successfully. The mean lateral size of GO, GO-POAA and GO-chitosan was calculated using the sum of the two longest lengths on the sheet and taking the average of those values to obtain the mean lateral size of the GO materials (Additional file 1: Figure S5).

### Culturing bacteria

*Escherichia coli* (ATCC 8739) and *B. subtilis* (ATCC 6633) were grown in either nutrient agar (per liter: microbiology powder 8 g, microbiology agar 12 g and tune pH to 7.0) or nutrient medium (per liter: microbiology powder 8 g and tune pH to 7.0) and incubated at 37 °C, respectively.

### Analysis of biocompatibility and antimicrobial ability for POAA and chitosan using the CFU counting method

Polyoxyalkyleneamine was dissolved in distilled water; and chitosan was dissolved with 0.5% (v/v) aqueous acetic acid and then the supernatant was added with 4N NaOH and the pH was adjusted to 7.0–7.5. The prepared POAA and chitosan solutions were added to bacteria (OD_600_ ~ 0.05) to achieve the final concentration of 0.01–100 μg mL^−1^, and then incubated with a nutrient medium for 3 h at 37 °C. The incubated bacteria were then diluted to a dilution factor of 10^−5^–10^−8^ and plated on the agar plates. The plates were incubated aerobically at 37 °C for 12–16 h. After incubation, the number of surviving bacteria was determined and is expressed as a percentage (%) that corresponds to the unit of CFU mL^−1^. Data are mean ± SD (n = 6).

### Growth curves for the bacteria

Graphene oxide, GO-POAA and GO-chitosan (GO materials were delivered in a dose of 50 μg mL^−1^) were added to bacteria (OD_600_ ~ 0.05), respectively, and incubated with either nutrient medium or PBS alone for 3 h at 37 °C. The absorbance of the optical density was recorded at 600 nm. Data are mean ± SD (n = 6).

### Analysis of the antimicrobial ability for GO, GO-POAA and GO-chitosan using a LIVE/DEAD kit and the CFU counting method [14, 35]

#### LIVE/DEAD kit

Graphene oxide, GO-POAA and GO-chitosan (GO materials were delivered in a dose of 50 μg mL^−1^) were added to bacteria (OD_600_ ~ 0.05) and incubated with either nutrient medium or PBS alone for 3 h at 37 °C, and then SYTO 9/propidium iodide (PI) (Live/Dead BacLight Bacterial viability Kits, Invitrogen) was then added and mixed. After incubation, the GO-, GO-POAA and GO-chitosan-treated-bacteria were centrifuged at 2000 rpm for 10 min, after which the supernatant was discarded, but the pellet was collected. The pellet was resuspended in nutrient medium and PBS, respectively, and centrifuged. This process was repeated for 3–5 times to wash out the nonspecific binding. The pellets were then stained using a using a LIVE (SYTO 9, as indicated by green fluorescence)/DEAD (PI, as indicated by red fluorescence) kit (L7012, Invitrogen, USA) in line with the manufacturer’s instructions. Images were acquired using fluorescence microscopy (OLYMPUS), and the quantification measurement for live or dead (%) was estimated and using fluorescence microscopy (F-2500, Hitachi, Japan).

#### CFU counting method

Graphene oxide, GO-POAA and GO-chitosan were added to bacteria (OD_600_ ~ 0.05), respectively, and incubated with either nutrient medium or PBS alone for 3 h at 37 °C. The GO-, GO-POAA and GO-POAA-treated-bacteria were then diluted to a dilution factor of 10^−5^–10^−8^ and plated on the agar plates. The plates were incubated aerobically at 37 °C for 12–16 h. After incubation, the number of surviving bacteria were determined using a CFU (CFU mL^−1^) counting assay and the unit is expressed as a percentage that corresponds to the unit of CFU mL^−1^. Data are mean ± SD (n = 6).

### ROS detection

#### Superoxide radical anion (O_2_^**·**−^)

Graphene oxide materials (up to 20 μm wide) were delivered at the doses of 10–300 μg mL^−1^. GO-, GO-POAA- and GO-chitosan-treated-bacteria (OD_600_ ~ 0.05) were respectively incubated with either nutrient medium or PBS alone for 3 h at 37 °C and then mixed and incubated with 1 mL 0.45 mM XTT for 5 h in the dark [7, 28]. XTT interacts with superoxide radical anion and form the XTT-formazan, which is strongly absorbed at a wavelength of 470 nm. The absorption was recorded using a UV–vis spectrometer (U-4100, Hitachi, Japan). Data are mean ± SD (n = 6).

#### Singlet oxygen (^1^O_2_)

GO materials (up to 20 μm wide) were delivered at the doses of 10–300 μg mL^−1^. GO-, GO-POAA- and GO-chitosan-treated-bacteria (OD_600_ ~ 0.05) were incubated with either nutrient medium or PBS alone for 3 h at 37 °C, and then 1 μM of Singlet Oxygen Sensor Green Reagent (Ex/Em: 488/525 nm) was added. Measurements were obtained using a fluorescence spectrophotometer (F-2500, Hitachi, Japan) in line with the manufacturer’s instructions [35]. Data are mean ± SD (n = 6).

#### Hydroxyl peroxide (H_2_O_2_)

GO materials (up to 20 μm wide) were delivered at concentrations of 10 and 100 μg mL^−1^, respectively. GO-, GO-POAA- and GO-chitosan-treated-bacteria (OD_600_ ~ 0.05) were incubated with either nutrient medium or PBS alone for 3 h at 37 °C. The bacteria were centrifuged (5000 rpm, 5 min) and pellet was mixed with diluted CM-H_2_DCFDA [29] solution (CM-H_2_DCFDA 2.5 μg + 99.5% ethanol 500 μL, and diluted 1000-fold with 1× PBS to a final volume of ~ 500 mL) for incubation at 37 °C for 10–20 min. Finally, 5 μL catalase (From Bovine Liver) was added to the reaction and the bacteria were incubated at 4 °C for flow cytometry measurements (BD, FACS 101, USA). The colorless CM-H_2_DCFDA passes through cell membranes and converts itself into 2′,7′-dichlorodihydrofluorescin (DCFH). In the presence of H_2_O_2_, DCFH is oxidized to dichlorodihydrofluorescein (DCF), which emits green fluorescence (Em: ~ 530 nm) at an intensity that is proportional to the intracellular H_2_O_2_ concentration. The signal was collected in FL-1 photomultiplier tubes (wavelength of filter: 515–545 nm).

#### GSH oxidation (O_2_^**·**−^) (the Ellman’s assay)

GO materials (up to 20 μm wide) were delivered at the doses of 10–100 μg mL^−1^. GO-, GO-POAA- and GO-chitosan-treated-bacteria (OD_600_ ~ 0.05) were incubated with either nutrient medium or PBS alone for 3 h at 37 °C and centrifuged and the pellets were collected. The pellets were mixed with 50 mM bicarbonate buffer (pH 8.6) and GSH/0.8 mM bicarbonate buffer) was added in the dark. This was then incubated in a shaker for 2 h at 37 °C. The following experiments were then performed, as in previous studies [15, 30]. Loss of GSH % = (absorbance of negative control − absorbance of sample)/absorbance of negative control × 100. Data are mean ± SD (n = 6). All of the data represent the same region and GO-treated-bacteria are compared to bacteria alone without treating any material (negative control). The higher the percentage exhibited, the greater is the oxidative stress.

### Statistical analysis

The statistical significance was calculated using an analysis of variance. The *p* value was considered statistically significant for all of the treatments.

## Additional file

**Additional file 1: Figure S1.** FTIR spectra. **Figure S2.** FTIR spectra. **Figure S3.** The oxidation of GSH by GO sheets and GO-based materials. **Figure S4.** The ROS assays. **Table S1.** Instruments with different functions are used to characterize materials. **Figure S5.** The mean lateral size.

## Abbreviations

GO: graphene oxide
POAA: polyoxyalkyleneamine
ROS: reactive oxygen species
PBS: phosphate buffered saline
TEM: transmission electron microscopy
AFM: atomic force microscopy
XRD: X-ray diffraction
XPS: X-ray photoelectron spectroscopy
UV-vis: ultraviolet-visible
FTIR: Fourier transform infrared
CFU: colony forming unit
*E. coli*: *Escherichia coli*
*B. subtilis*: *Bacillus subtilis*
O_2_^**·**−^: superoxide radical anion
^1^O_2_: singlet oxygen
H_2_O_2_: hydrogen peroxide
XTT: 2, 3-bis (2-methoxy-4-nitro-5-sulfophenyl)-2H-tetrazolium-5-carboxanilide
GSH: glutathione (γ-l-glutamyl-l-cysteinyl-glycine, gsh
CM-H_2_DCFDA: 5-(and 6-)chloromethyl-2’,7′-dichlorodihydrofluorescein diacetate
PI: propidium iodide
DCFH: 2′,7′-dichlorodihydrofluorescin
DCF: dichlorodihydrofluorescein
