# Supplementary material for: Graphene oxide conjugated with polymers: a study of culture condition to determine whether a bacterial growth stimulant or an antimicrobial agent?
Source: J Nanobiotechnology. 2018 Jan 10;16:1. doi: 10.1186/s12951-017-0328-8 (PMC5761102; doi:10.1186/s12951-017-0328-8)
Supplement: Supplementary file 1 — Additional file 1: Figure S1. FTIR spectra. Figure S2. FTIR spectra. Figure S3. The oxidation of GSH by GO sheets and GO-based materials. Figure S4. The ROS assays. Table S1. Instruments with different functions are used to characterize materials. Figure S5. The mean lateral size. [file 12951_2017_328_MOESM1_ESM.pdf]

Additional file

Graphene Oxide Conjugated with Polymers: a Study of Culture Condition to Determine Whether a Bacterial Growth Stimulant or an Antimicrobial Agent?

Ping-Ching Wu[1], Hua-Han Chen[2], Shih-Yao Chen[3], Wen-Lung Wang[3], Kun-Lin Yang[4], Chia-Hung Huang[5,6], Hui-Fang Kao[7], Jui-Cheng Chang[8], Chih-Li Lilian Hsu[9], and Jiu-Yao Wang[9,10*], Ting-Mao Chou[11*], Wen-Shuo Kuo[10,12,13*]

[1]*Department of Biomedical Engineering, National Cheng Kung University, Tainan 701, Taiwan (R.O.C.).*

[2]*Department of Food Science, National Penghu University of Science and Technology, Penghu 880, Taiwan (R.O.C).*

[3]*Department of Internal Medicine, National Cheng Kung University Hospital, College of Medicine, National Cheng Kung University, Tainan 701, Taiwan (R.O.C.).*

[4]*Athena Institute of Holistic Wellness, Wuyishan 354300, Fujian, China.*

[5]*Metal Industries Research & Development Centre, Kaohsiung 811, Taiwan (R.O.C.).*

[6]*Department of Materials Science Engineering, National Cheng Kung University, Tainan 701, Taiwan (R.O.C.).*

[7]*Department of Nursing, National Tainan Junior College of Nursing, Tainan 700, Taiwan (R.O.C.).*

[8]*Department of Chemical Engineering, National Cheng Kung University, Tainan 701, Taiwan (R.O.C.).*

[9]*Department of Microbiology & Immunology, National Cheng Kung University Hospital, College of Medicine, National Cheng Kung University, Tainan 701, Taiwan (R.O.C.).*

[10]*Department of Pediatrics, National Cheng Kung University Hospital, College of Medicine, National Cheng Kung University, Tainan 701, Taiwan (R.O.C.).*

[11]*Division of Plastic Surgery, Department of Surgery, E-Da Hospital, Kaohsiung 824, Taiwan (R.O.C.).*

[12]Center for Micro/Nano Science and Technology, National Cheng Kung University, Tainan 701, Taiwan (R.O.C.).

[13]Advanced Optoelectronic Technology Center, National Cheng Kung University, Tainan 701, Taiwan (R.O.C.).

E-mail: wbcxyz@gmail.com (Ping-Ching Wu); huahan@gms.npu.edu.tw (Hua-Han Chen); z9903038@email.ncku.edu.tw (Shih-Yao Chen);

n116699@mail.hosp.ncku.edu.tw (Wen-Lung Wang); coolinyang@gmail.com (Kun-Lin Yang); chiahung@mail.mirdc.org.tw (Chia-Hung Huang); kaohuif@gmail.com (Hui-Fang Kao); d700215@gmail.com (Jui-Cheng Chang); lilianlh@mail.ncku.edu.tw (Chih-Li Lilian Hsu).

*Correspondence: Jiu-Yao Wang (Email: a122@mail.ncku.edu.tw) and Wen-Shuo Kuo (Email: wenshuokuo@mail.ncku.edu.tw). National Cheng Kung University Hospital, College of Medicine, National Cheng Kung University, Tainan 701, Taiwan (R.O.C.); Ting-Mao Chou (Email: ed109842@edah.org.tw). E-Da Hospital, Kaohsiung 824, Taiwan (R.O.C.).

Fourier transform infrared (FTIR) spectroscopy was also used to analyze the exposed functional groups of GO and GO-based materials (**Figs. S1-S2**). **Fig. S1** shows the characteristic bands of graphite around 694 cm^{-1} for plane C=C bending, 1450 cm^{-1} and 1740 cm^{-1} for C=C ring stretching; the characteristic bands of POAA around 614 cm^{-1} for broad N-H out-of-plane bending, 972 cm^{-1} and 1110 cm^{-1} for C-O stretching, 1280 cm^{-1} and 1360 cm^{-1} for C-H bending, 1660 cm^{-1} for water H-O-H bending. Furthermore, the characteristic bands of chitosan around 670 cm^{-1} for broad N-H out-of-plane bending, 910 cm^{-1} for O-H out-of-plane bending, 1080 cm^{-1} and 1110 cm^{-1} for C-O stretching, 1280 cm^{-1},1340 cm^{-1} and 1390 cm^{-1} for C-H bending, 1670 cm^{-1} for water H-O-H bending, 2706 cm^{-1} for C-H stretching and N-H stretching. Furthermore, **Fig. S2** shows the characteristic bands of GO around 670 cm^{-1} for plane C=C bending, 852 cm^{-1} and 887 cm^{-1} for the aromatic C-H deformation, 1010 cm^{-1} for epoxy stretching, 1070 cm^{-1} and 1195 cm^{-1} for C-O stretching, 1330 cm^{-1} for tertiary alcoholic C-OH bending, 1400 cm^{-1} for symmetric carboxylate $C(=O)_2^-$ stretching, 1640 cm^{-1} for C=C ring stretching and water H-O-H bending, and 1740 cm^{-1} for C=O stretching. The epoxy and tertiary alcohol groups play an increasing role in the basal interlayer distance and in the hydrophilicity of the compound. In the periphery, the appearance of phenolic OH and ketone signals indicates the presence of phenol and carboxylic acid [1]. The broad absorption at 3000~3600 cm^{-1} for O-H stretching vibrations is partially related to liquid water since the H-O-H bending band of the H_2O molecules was also observed at 1640 cm^{-1}. Previous studies have reported that complete water removal from GO is practically impossible [2]. After conjugation of POAA, characteristic bands of GO-POAA around 611 cm^{-1} and 683 cm^{-1} for broad N-H out-of-plane bending, 845 cm^{-1} for the aromatic C-H deformation, 966 and 1120 cm^{-1} for C-O stretching, 1260 cm^{-1},1320 cm^{-1} and 1370 cm^{-1} for C-H bending, 1330 cm^{-1} for tertiary alcoholic C-OH bending, 1470 and 1660 cm^{-1} for C=C ring stretching, 1680 cm^{-1} for H-O-H bending, and 2880 cm^{-1} for N-H stretching. Furthermore, characteristic bands of GO-chitosan around 670 cm^{-1} for plane C=C bending, 677 cm^{-1} for broad N-H out-of-plane bending, 912 cm^{-1} for O-H out-of-plane bending, 1083 cm^{-1} and 1170 cm^{-1} for C-O stretching, 1270 cm^{-1}, 1320 cm^{-1} and 1400 cm^{-1} for C-H bending, 1320 cm^{-1} for tertiary alcoholic C-OH bending, 1650 cm^{-1} for C=C ring stretching and water H-O-H bending, and 2890 cm^{-1} for N-H stretching. After the characterization of FTIR, we confirmed that the GO had been successfully synthesized and well-decorated with POAA and chitosan, respectively.

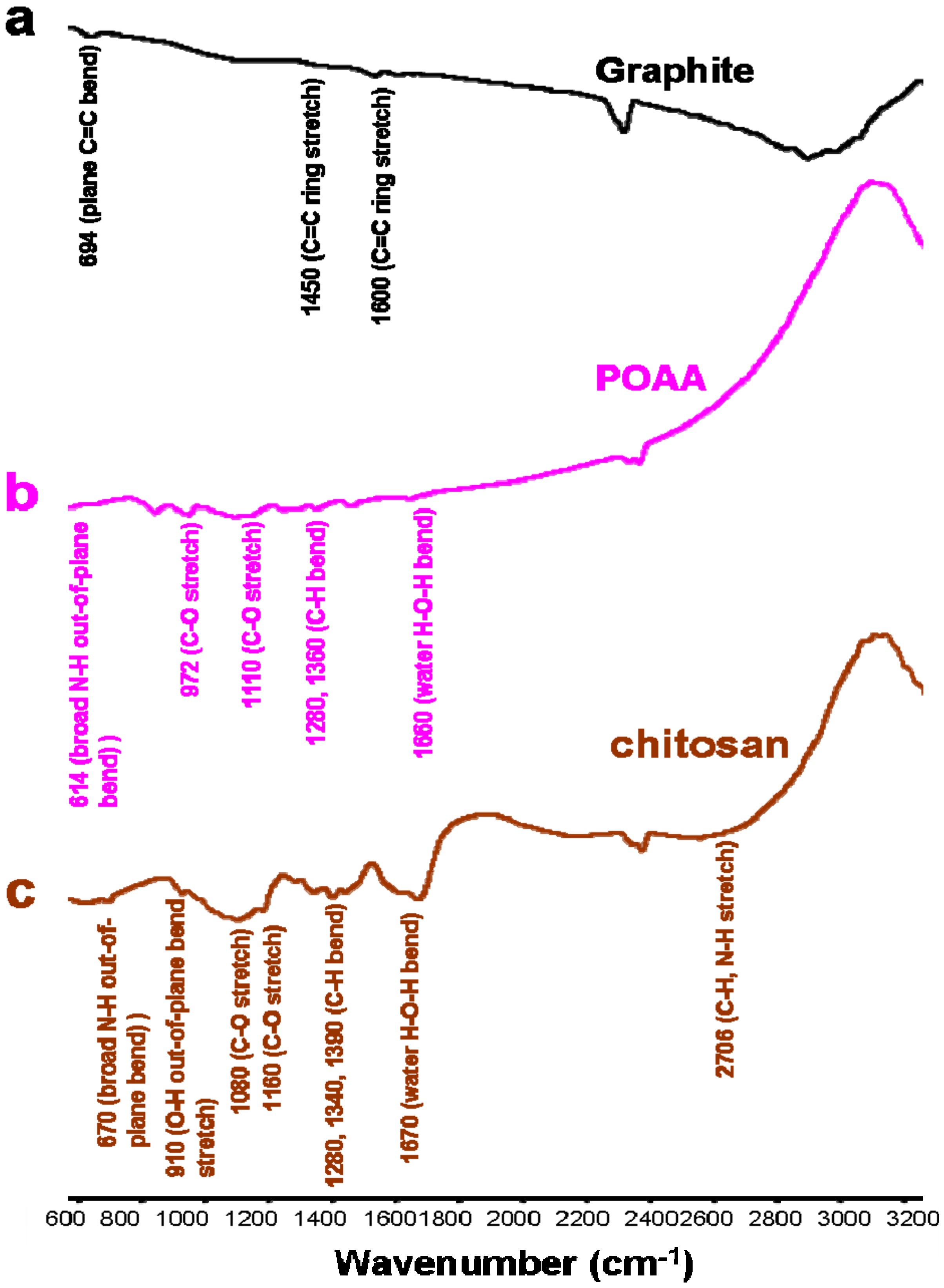

Fig. S1 FTIR spectra. (a) graphite, (b) POAA and (c) chitosan, respectively.

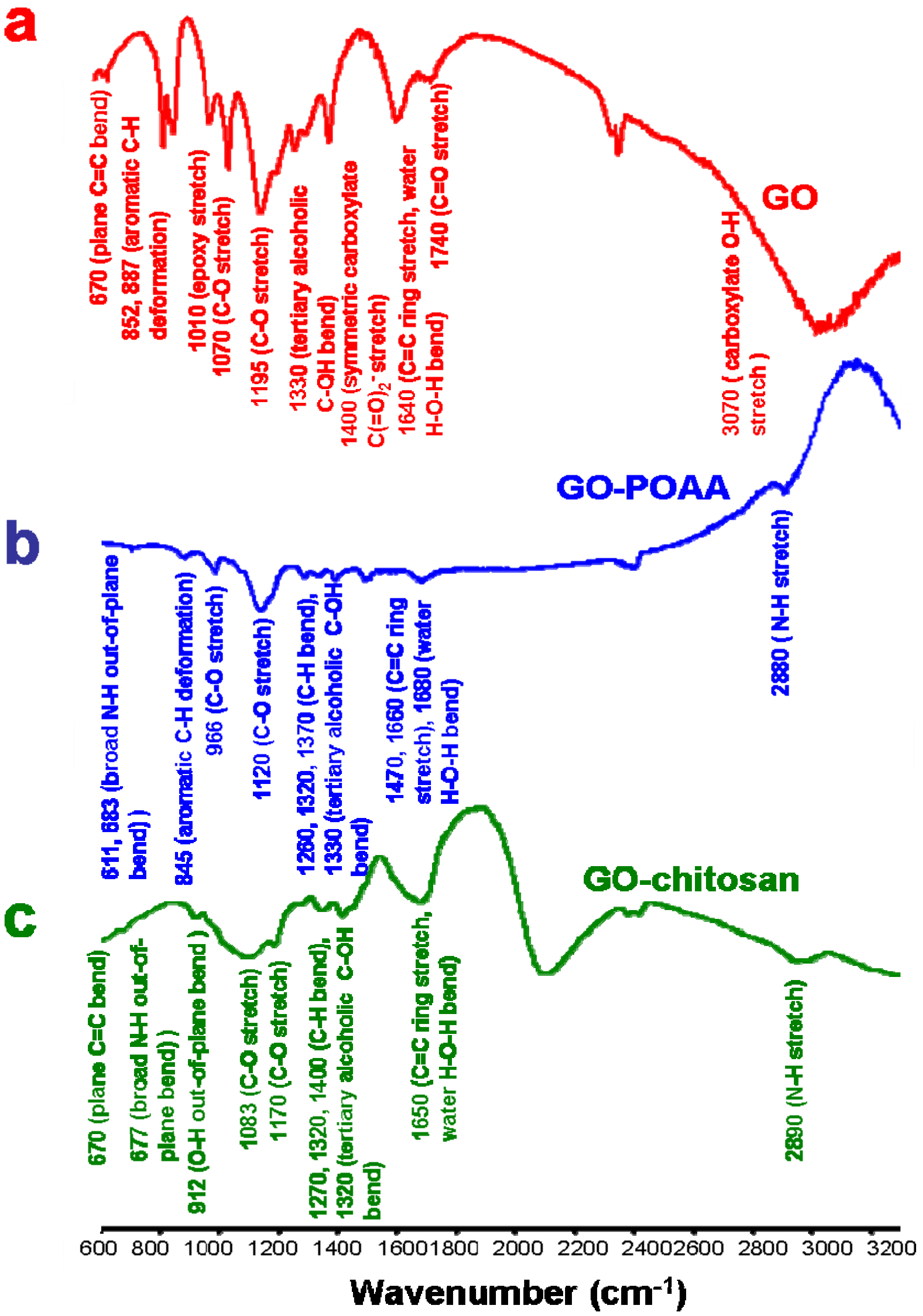

Fig. S2 FTIR spectra. (a) GO, (b) GO-POAA and (c) GO-chitosan, respectively.

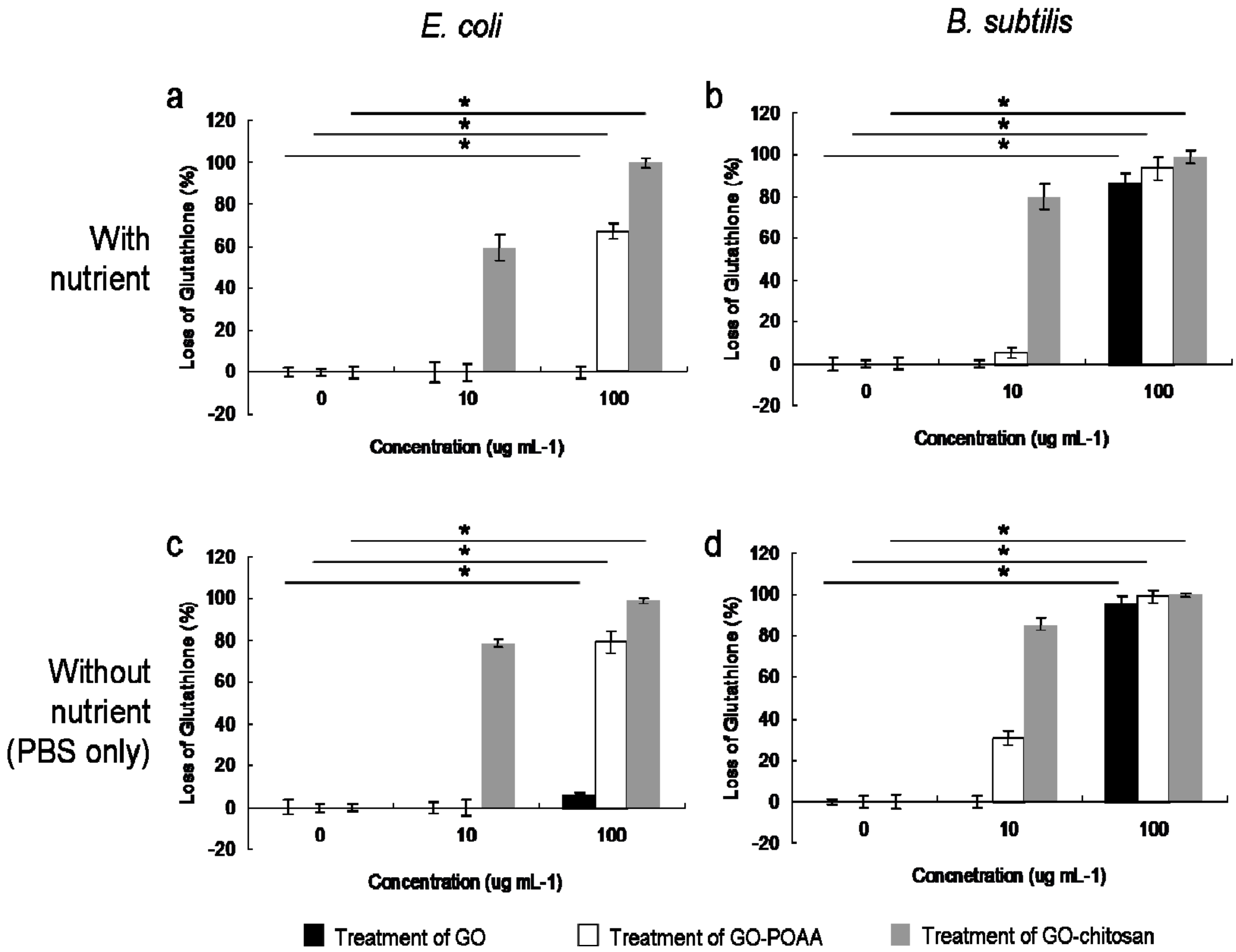

Fig. S3 The oxidation of GSH by GO sheets and GO-based materials. (a,c) *E. coli* and (b,d) *B. subtilis* were treated with GO, GO-POAA, and GO-chitosan (up to 20 μm wide) in either (a,b) nutrient or (c,d) PBS alone. Negative control (0 μg mL^{-1}): bacteria alone without any treatment. For *E. coli*, (a) p= 0.996, p< 0.001 and p< 0.0001, (c) p= 0.231, p< 0.001 and p< 0.0001 are for treatment of GO, GO-POAA and GO-chitosan, respectively; for *B. subtilis,* (b) p< 0.0001, p< 0.0001 and p< 0.0001, (d) p< 0.0001, p< 0.0001 and p< 0.0001 are for treatment of GO, GO-POAA and GO-chitosan, respectively. Data are means ± SD (n = 6). *p value obtained by Student's *t* test.

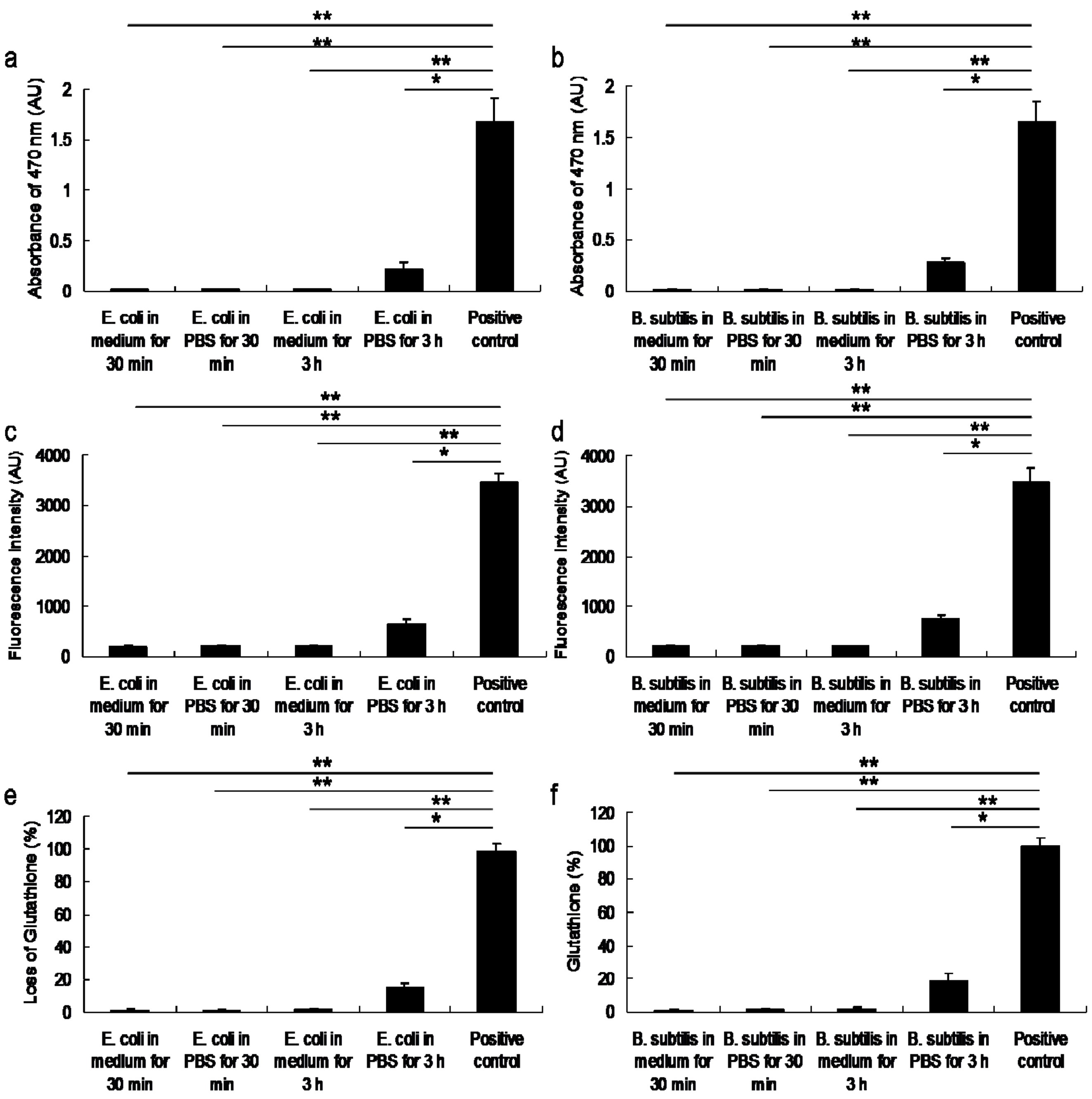

Fig. S4 The ROS assays. The generated superoxide radical anion ($O_2^{\cdot -}$) of (a) *E. coli* or (b) *B. subtilis* alone was in either medium or PBS at 37 °C for 30 min or 3 h, respectively. XTT was used to monitor the generated $O_2^{\cdot -}$ and the absorbance at 470 nm was recorded. For *E. coli*, (a) **$p < 0.0001$ and *$p = 0.0077$; for *B. subtilis,* (b) **$p < 0.0001$ and *$p = 0.0092$. The generated singlet oxygen ($^1O_2$) of (c) *E. coli* or (d) *B. subtilis* alone was under the same procedure. Singlet Oxygen Sensor Green Reagent was used to monitor the generated $^1O_2$ with a fluorescence spectrophotometer (Ex/Em: 488/525 nm). For *E. coli*, (c) **$p < 0.0001$ and *$p = 0.0115$; for *B. subtilis,* (d) **$p < 0.0001$ and *$p = 0.0174$. Using the Ellman's assay to monitor the oxidation of

GSH for (e) *E. coli* or (f) *B. subtilis* alone which was under the same procedure. For *E. coli*, (e) **p< 0.0001 and *p= 0.0096; for *B. subtilis,* (f) **p< 0.0001 and *p= 0.0131. Positive control: with the treatment of 50 μM tert-butyl hydroperoxide (TBHP) [3]. Data are means ± SD (n = 6). *p value obtained by Student's *t* test.

Experimental procedure. For $O_2^{\cdot -}$ detection, bacteria alone (OD_{600}~ 0.05) were respectively incubated with either nutrient medium or PBS alone for 30 min or 3 h at 37 °C and then mixed and incubated with 1 mL 0.45 mM XTT for 5 h in the dark. The absorption (a wavelength of 470 nm) was recorded using a UV-vis spectrometer. For $^1O_2$ detection, bacteria alone (OD_{600}~ 0.05) were respectively incubated with either nutrient medium or PBS alone for 30 min or 3 h at 37 °C and then 1 μM of Singlet Oxygen Sensor Green Reagent (Ex/Em: 488/525 nm) was added. Measurements were obtained using a fluorescence spectrophotometer. For the oxidation of GSH, bacteria alone (OD_{600}~ 0.05) were respectively incubated with either nutrient medium or PBS alone for 30 min or 3 h at 37 °C and centrifuged and the pellets were collected. The pellets were mixed with 50 mM bicarbonate buffer (pH 8.6) and GSH/0.8mM bicarbonate buffer) was added in the dark. This was then incubated in a shaker for 2 h at 37 °C. Loss of GSH % was calculated according to the part of Methods in the manuscript. For positive control, the ROS was recorded following the procedure that the individual detection reagent of ROS was mixed with 50 μM TBHP. Data are means ± SD (n = 6).

Table. S1 Instruments with different functions are used to characterize materials.

Instrument	Function
TEM	Morphology, interlayer spacing and lateral size
AFM	Thickness
Zeta potential	Surface charge
XRD	Crystallinity
Raman	Crystallinity
XPS	Surface chemistry
UV-vis	Surface plasma
FTIR	Functional group of surface

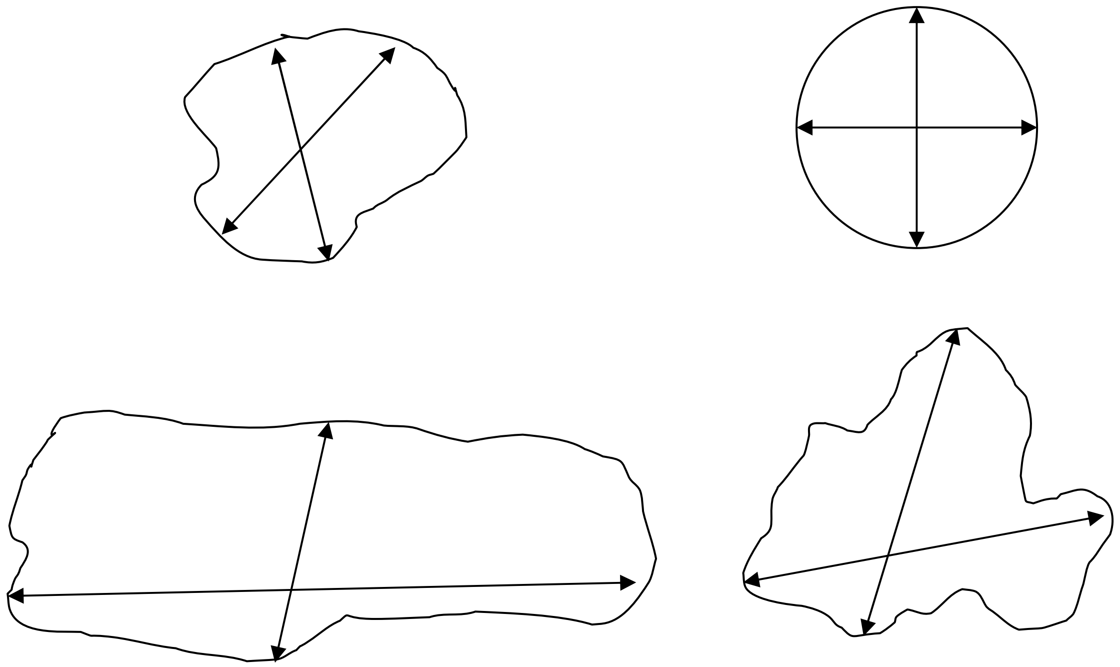

Fig. S5 The mean lateral size. The sum of the two longest lengths on the sheet is averaged to obtain the mean lateral size of the GO materials.

Reference

1. Gao W, Alemany LB, Ci L, Ajayan PM. New insights into the structure and reduction of graphite oxide. Nat. Chem. 2009; 1: 403-8.

2. Xie G, Wang Z, Cui Z, Shi Y. Ni-Fe-Co-P coatings on coiled carbon nanofibers. Carbon. 2005; 43: 3181-3.

3. Possel H, Noack H, Augustin W, Keilhoff G, Wolf G.. 2,7-Dihydrodichlorofluorescein diacetate as a fluorescent Marker for Peroxynitrite Formation. FEBS Lett 1997; 416: 175-8.
